# Profiling the transcription factor regulatory networks of human cell types

**DOI:** 10.1093/nar/gku923

**Published:** 2014-10-09

**Authors:** Shihua Zhang, Dechao Tian, Ngoc Hieu Tran, Kwok Pui Choi, Louxin Zhang

**Affiliations:** 1 National Center for Mathematics and Interdisciplinary Sciences, Academy of Mathematics and Systems Science, Chinese Academy of Sciences, Beijing 100190, China; 2 Department of Statistics and Applied Probability, National University of Singapore, Singapore 117546, Singapore; 3 Division of Mathematical Sciences, Nanyang Technological University, Singapore 637371, Singapore; 4 Department of Mathematics, National University of Singapore, Singapore 119076, Singapore; 5 National University of Singapore Graduate School for Integrative Sciences and Engineering, Singapore 117456, Singapore

## Abstract

Neph *et al.* (2012) (Circuitry and dynamics of human transcription factor regulatory networks. *Cell*, 150: 1274–1286) reported the transcription factor (TF) regulatory networks of 41 human cell types using the DNaseI footprinting technique. This provides a valuable resource for uncovering regulation principles in different human cells. In this paper, the architectures of the 41 regulatory networks and the distributions of housekeeping and specific regulatory interactions are investigated. The TF regulatory networks of different human cell types demonstrate similar global three-layer (top, core and bottom) hierarchical architectures, which are greatly different from the yeast TF regulatory network. However, they have distinguishable local organizations, as suggested by the fact that wiring patterns of only a few TFs are enough to distinguish cell identities. The TF regulatory network of human embryonic stem cells (hESCs) is dense and enriched with interactions that are unseen in the networks of other cell types. The examination of specific regulatory interactions suggests that specific interactions play important roles in hESCs.

## INTRODUCTION

Living cells are the products of transcription programs involving thousands of genes. Sequence-specific transcription factor (TF) proteins regulate target genes by binding to promoter regions adjacent to the DNA sequences of the genes. There are less than 2000 TFs in the human genome (1–4). They work cooperatively to enhance or inhibit their target genes to achieve high specificity, and thus to precisely control the condition-dependent expression of the genes to respond to extracellular stimuli. Hence, the mutual interactions among TFs determine cellular identity and shape complex cellular functions (5,6). This makes the study of human TFs on a system-wide scale of vitally important (7). In systems biology, regulatory interactions among TFs are modeled as a TF regulatory network in which the nodes are the TFs and the links represent the regulatory relationship among TFs.

Over the past decade, a great deal of information on the organization of regulatory interactions has been obtained particularly for *Escherichia coli* and *Saccharomyces cerevisiae* (8–12). However, comprehensive generation of cell-type regulatory interactions for humans has been a challenge. First, there are a large number of human TFs as mentioned above, but the data collected from individual experiments often target one cell type and only a few TFs in a particular condition (13–15). Second, correlation-based analyses of microarray gene expression data often do not capture the orientation of transcriptional regulations, a necessity for deep analyses of regulatory interactions (16,17). Fortunately, the genome-wide DNaseI footprinting technique has recently been adopted to determine the regulatory interactions of sequence-specific TFs in the 41 human cell types (18). This provides a valuable resource for deciphering regulatory mechanisms in different human cells.

The TF regulatory networks for *E. coli* (19), *S. cerevisiae* (19,20), mice (21) and humans (12) exhibit hierarchical organizations. Most importantly, these organizations interplay with TF dynamics (19,20). In the present paper, we investigate the structural organizations and dynamics of the 41 human cell-type TF regulatory networks reported in (18) using the vertex-sort algorithm developed in Jothi *et al.* (20). Our findings are interpreted to indicate three insightful conclusions. First, the human cell-type TF regulatory networks share similar global three-layer (top, core and bottom) hierarchical architectures, which are markedly different from that of the yeast TF regulatory network. On the other hand, there are significant differences in the TF regulatory interactions among cell types, as suggested by our finding that wirings around a few TFs can distinguish cell identities well. Second, the TF regulatory network of the human embryonic stem cell (hESC) is dense and has different topological properties from all the other networks. Finally, there are more specific regulatory interactions than thought in the hESCs. These cell-type regulatory interactions and the TFs involved may play unique roles in maintaining pluripotency.

## MATERIALS AND METHODS

### Network data

The TF regulatory networks of 41 human cell types have been taken from recent work by Neph *et al.* (18). These networks were derived from the DNaseI footprinting data and the predicted TRANSFAC motif-binding sites. Each network contains about 475 TFs and 11200 interactions.

According to the physiological and functional properties, Neph *et al.* (18) divided the 41 cell types into eight classes: blood (seven cell types), cancer (two cell types), endothelia (four cell types), epithelia (six cell types), ESCs (one cell type), fetal (three cell types), stroma (14 cell types) and viscera (four cell types).

### Discovery of the hierarchical structures of the regulatory networks

We used the vertex-sort algorithm (20) to identify the hierarchical structure of a regulatory network. The vertex-sort algorithm first collapses strongly connected components into supernodes to form a directed acyclic graph, and then constructs its transposed graph by reversing the directions of the edges. Next, it uses the topological structures of both the directed acyclic graph and its transposed graph to classify the original nodes into the top, core and bottom layers.

### Classifying cell types based on TF regulatory networks

Neph *et al.* (18) made use of the connectivity of the TF regulatory networks to classify the 41 human cell types. Specifically, they computed all the pairwise Euclidean distances between the normalized node-degree (NND) vectors of the networks, and then applied the Ward clustering method (22) to cluster the cell types.

Instead, we used local connectivity, defined by a subset of nodes in the networks, to classify the cell types. Given a small set of TFs, *A*, we define the feature vector of each cell type to be $(x_1 , \ldots ,x_n )$, where *n* is the number of TFs in the corresponding network and where *x_i_* = 1 if the *i*th TF is a target of some TFs in *A* and 0 otherwise. Principal component analysis was then applied to the feature vectors to reduce the dimension and the noise of feature vector data. We computed the pairwise Euclidean distances based on the first seven principal components of the 41 feature vectors and then applied Ward clustering to classify the cell types.

### Measuring the accuracy of the classifications of cell types

The Rand Index (RI) (23) was used to assess the quality of cell type classifications. To this end, the 41 cell types are partitioned into four categories: (i) stromal and epithelial, (ii) blood, (iii) endothelial and (iv) cancer, ESC, and fetal tissues.

### Detection of regulatory complex-target modules in hESCs

The hESC-specific interactions are interactions that are only found in the regulatory network of hESCs. A total of 1509 interactions were identified (Supplementary Table S1).

We used these interactions to identify regulatory complex-target modules that are specific to hESCs. For a protein complex, *C*, and a set of TFs, *B*, we say that *C* and *B* form a regulatory complex-target module if *C* contains two or more TFs such that all TFs in *B* are regulated by every TF (in *C*) only in the hESCs. We detected 55 regulatory complex-target modules (Supplementary Table S2) using the protein complexes reported in (24).

### Comparing two distributions

The Wilcoxon rank-sum test was used to determine whether the RI was significantly higher when grouping 41 cell types based on the targets of a few TFs compared to random grouping.

The gene expression data of 79 human tissues (25) were used to investigate whether a TF gene was stably expressed across tissues. The deviation of an expression level from being a constant is measured in terms of its relative entropy (also known as Kullback–Leibler divergence). In our context, for a gene, it is computed as $\log _2 79 + \sum\nolimits_j {f_j \log _2 (f_j )}$, where $f_j = e_j /\left( {\sum\nolimits_{k = 1}^{79} {e_k } } \right)$ and *e_j_* is the expression level of the gene in tissue *j* (1). The entropy equals 0 if the gene expression levels are identical in all 79 tissues. The Wilcoxon rank-sum test was also used to test whether the TFs involved in housekeeping (HK) interactions were more stably expressed than the other TFs.

## RESULTS

### Wirings around a few TFs are enough to distinguish cell identities

**Figure 1.**
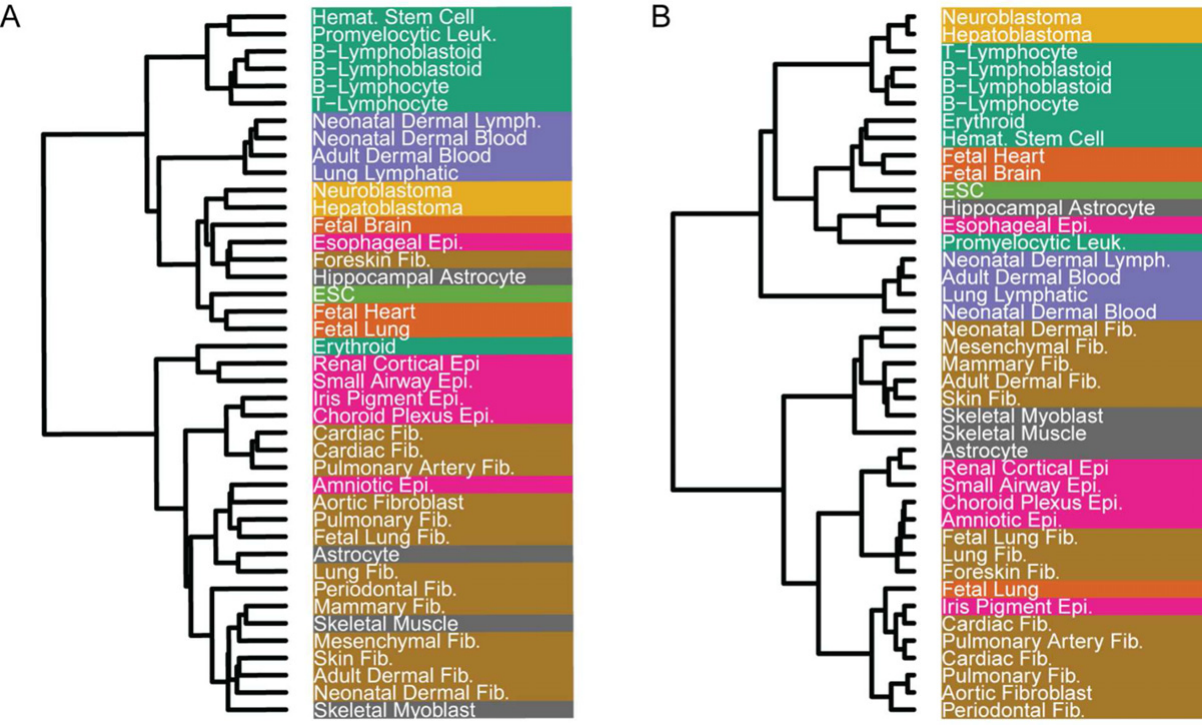
The hierarchical clustering of 41 cell types, where the color indicates which classes they belong to (Methods). (**A**) The clustering reported in (18) and redrawn for the purpose of comparison, which is based on the pairwise Euclidean distances between the NND vectors of the corresponding TF regulatory networks, has RI = 0.801. (**B**) Our clustering, which is based on the distribution of the downstream targets of the seven signal transducer and activator of transcription (STAT) proteins, has RI = 0.856.

Neph *et al.* (18) made use of the global connectivity of the TF regulatory networks to classify the 41 human cell types (Methods). The resulting grouping (redrawn in Figure 1A) strikingly groups the anatomical and functional cell-type groups into clearly preannotated classes with RI = 0.801. Surprisingly, the local connection patterns involving five to nine arbitrarily selected TFs are also good enough to obtain comparable classifications with the RI being in the range from 0.7 to 0.9 on average (Methods, Supplementary Figure S1).

**Figure 2.**
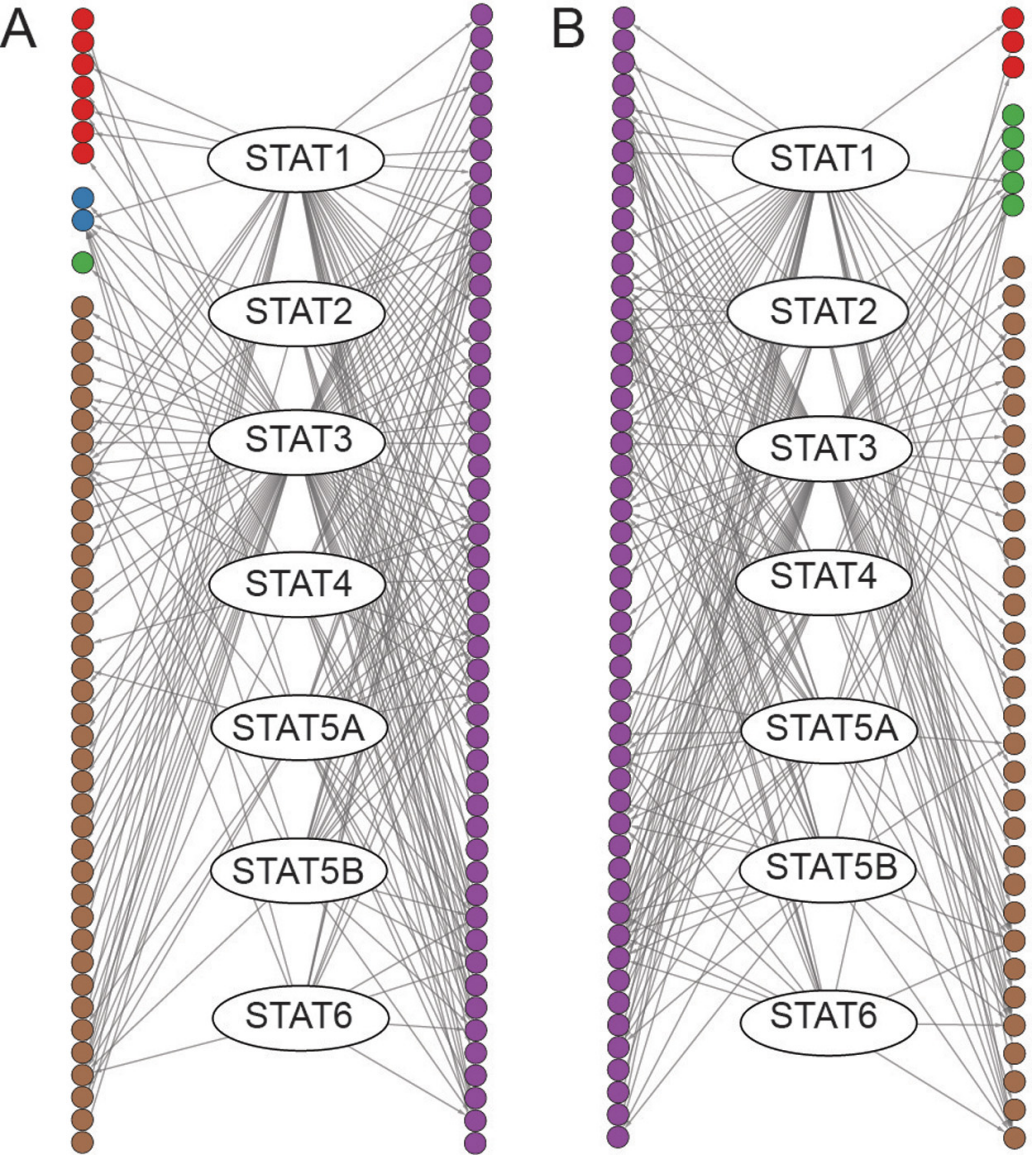
The STATs and their downstream regulatory targets in hESCs (**A**) and HSCs (**B**). Purple TFs are those regulated by some STATs in both cell types. The cell fate commitment process (GO:0045165) is enriched in the targets of STATs in hESCs (Benjamini corrected *P*-value = 2.72e−7). Dark red and blue targets are the TFs annotated with the GO term. The hemopoietic or lymphoid organ development process (GO:0048534) is enriched in the targets of STATs in HSCs (Benjamini corrected *P*-value = 0.03). Green and blue targets are the TFs annotated with this GO term. Brown targets are other targets whose GO annotations are not given.

Let us consider the seven mammalian signal transducer and activator of transcription (STAT) proteins. The activation of STATs by the Janus kinase proteins serves as an alternative to the second messenger system, transmitting extracellular signals from a wide spectrum of cytokines, growth factors and other polypeptide ligands to the nuclei (26,27). A close examination finds that the TFs regulated by the STATs are annotated with different gene ontology (GO) terms in different regulatory networks. For example, as illustrated in Figure 2, TFs that are regulated by STATs in hESCs but not in hematopoietic stem cells (HSCs) are enriched in GO:0045165 (cell fate commitment, Benjamini corrected *P*-value = 2.72e−7). By contrast, TFs that are regulated by STATs in HSCs but not in hESCs are enriched in GO:0048534 (hemopoietic or lymphoid organ development, Benjamini corrected *P*-value = 0.03).

The diversity of the downstream TFs of the STATs might indicate their strong distinguishability for the classification of human cell types. Indeed, using the information on how the STAT proteins connect with their targets to classify the cell types, we obtained a grouping with RI = 0.856 (Figure 1B), which is even higher than the RI of the grouping of Neph *et al.* mentioned above.

### The hierarchical structures of 41 cell-type regulatory networks

The *E. coli*, yeast, rat, mouse and human regulatory networks all exhibit hierarchical organization (12,19–21). We investigate the hierarchical organization of the 41 human cell type networks using the vertex-sort algorithm (20).

**Figure 3.**
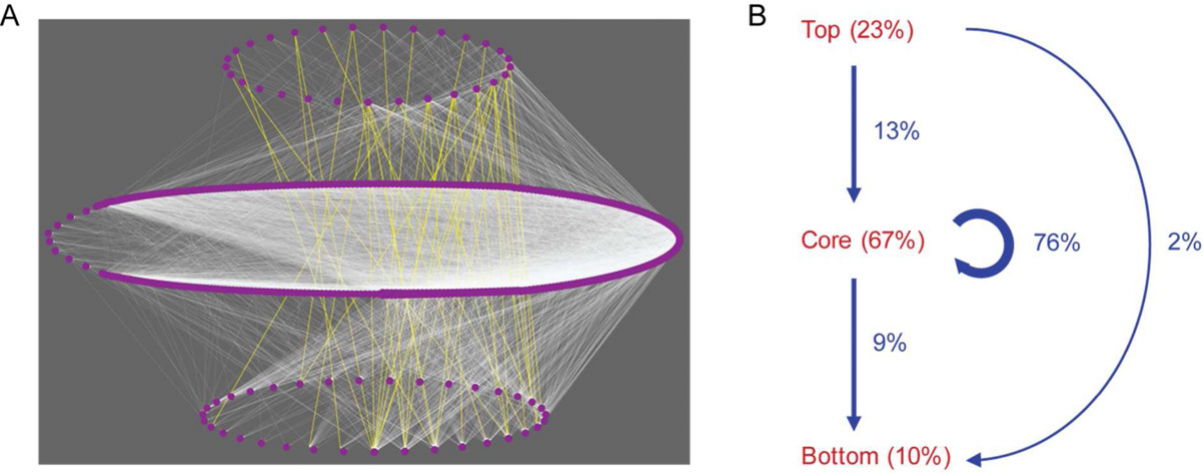
(**A**) A schematic view of the three-layer hierarchical structure of the hESC TF regulatory network. The links between the top and bottom layers are colored yellow. (**B**) A summary of average percentages of nodes (dark red) in the three layers and of links (blue) within and across the top, core and bottom layers in a human cell-type TF regulatory network.

For each network, the vertex-sort algorithm partitioned its nodes into the top, core and bottom layers (Figure 3A) (Methods). The percentages of TFs in the three layers of the 41 regulatory networks are reported in Supplementary Table S3. On average, 23% of TFs are classified into the top layer, 67% into the core layer and the lowest amount of TFs (10%) into the bottom layer (Figure 3B). The top, core and bottom layers of the 41 networks have 1 (i.e. HNF4G), 141 and 15 TFs in common, respectively.

When compared to the regulatory networks of other cell types, the hESC TF regulatory network has a significantly low number of TFs in the top layer (6%, *P*-value < 0.01, one-tailed test) and its core layer contains a significantly high number of TFs (85%, *P*-value < 0.01, one-tailed test). However, its bottom layer has a size (9%) similar to those of the other cell type networks (Supplementary Table S3).

To measure the degree of hierarchy in the three-layer structures obtained above, we calculated the local reaching centrality (LRC) of TFs in each of the 41 networks (28). As expected, the LRC of each TF in a layer is always greater than that of each TF in the layers below it in all except two stromal (HCF and HCM) networks. In the HCF network, only HOXC9 and NKX2-1 in the top layer have an extremely low LRC, smaller than the LRC of the TFs in the core layer. In the HCM network, only HOXC9 and NKX6-1 in the top layer have smaller LRC than that of TFs in the core layer. The mean values of the LRC of the TFs in a layer in the 41 regulatory networks are given in Supplementary Table S4. The global reaching centrality (GRC) of the 41 regulatory networks ranges from 0.065 to 0.125. Low GRC for each network is due to (i) there are only three hierarchical layers, (ii) the core layer is much larger than the top layers (67 versus 23% on average) and (iii) the LRC of a TF is slightly smaller in the core layer than in the top layer. These facts lead to the distribution of LRCs skew to the maximum LRC resulting in small GRC.

#### Distributions of network links

Seventy-six percent of links are distributed within the core layer (Supplementary Table S3 and Figure 3B). Both the size of the core layers and the links within them reveal the complex regulatory relationships among TFs in different human cells. The remaining links are distributed as follows: top → core (13%), top → bottom (2%) and core → bottom (9%), suggesting that TFs in the top layer mainly regulate TFs in the core layer.

#### Distributions of hubs

**Figure 4.**
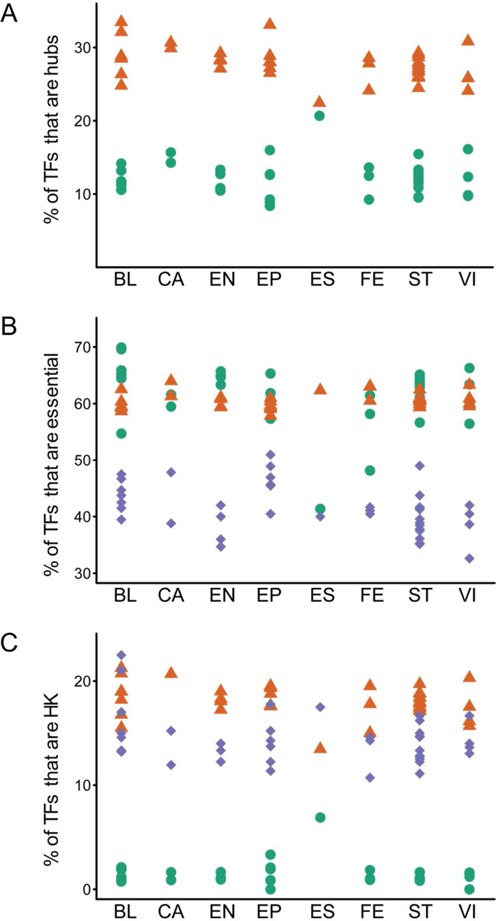
Percentages of TFs that are hubs (**A**), essential (**B**) and HK (**C**) in the top (green circle), core (brown triangle) and bottom (blue diamond) layers in 41 human cell-type TF regulatory networks, grouped according to cell class. Abbreviations: BL, blood; CA, cancer; EN, endothelia; EP, epithelia; ES, ESC; FE, fetal; ST, stromal cells; VI, visceral cells.

TFs with high out-degrees are crucial in that they have a large number of downstream targets. Following Jothi *et al.* (20), the top 20% TFs with the largest out-degree are defined as hubs in a regulatory network. There are 96–98 hubs that regulate at least 21 TFs in each of the 41 cell-type regulatory networks. The core layers of the networks are all enriched in hubs (all *P*-values ≤ 0.005, hypergeometric test, Figure 4A). All the top layers are depleted in hubs (all *P*-values ≤ 0.05, hypergeometric test) except in the networks of hESCs, HSCs, hippocampal astrocytes and mammary fibroblasts (Figure 4A). These results on hub enrichment are concordant with those of the yeast transcription network (20).

#### Distributions of essential TFs

Essential proteins are necessary for performing basic developmental functions. If they are disrupted, they will cause pre- or neonatal lethality (29). There are 280 essential TFs in each of the 41 networks. For each network, the percentages of essential proteins in the top and core layers are about the same (average difference ∼1%) (Figure 4B). By contrast, the percentage of essential proteins in the top layer (∼12%) is higher than in the core layer (∼6%) and in the bottom layer (∼3%) in the yeast transcription network (20).

#### Distributions of HK TFs

Here TFs encoded by HK genes (30) are called HK TFs. There are 63 HK TFs in each of the 41 networks. There are two, 54 and seven HK TFs, respectively, in the top, core and bottom layers of the hESC TF regulatory network. In the remaining 40 networks, all the core layers are enriched, whereas all the top layers are depleted in HK TFs (Figure 4C).

### HK and specific regulatory interactions

In analogy to genes, some regulatory interactions appear in only certain cell types, whereas many others are found in all cell types. Regulatory interactions that are only found in one cell type are called specific interactions; those that are found in all cell types are called HK interactions. Identifying the regulatory interactions belonging to the classes provides important biological insights into complex biological systems (31–34).

**Figure 5.**
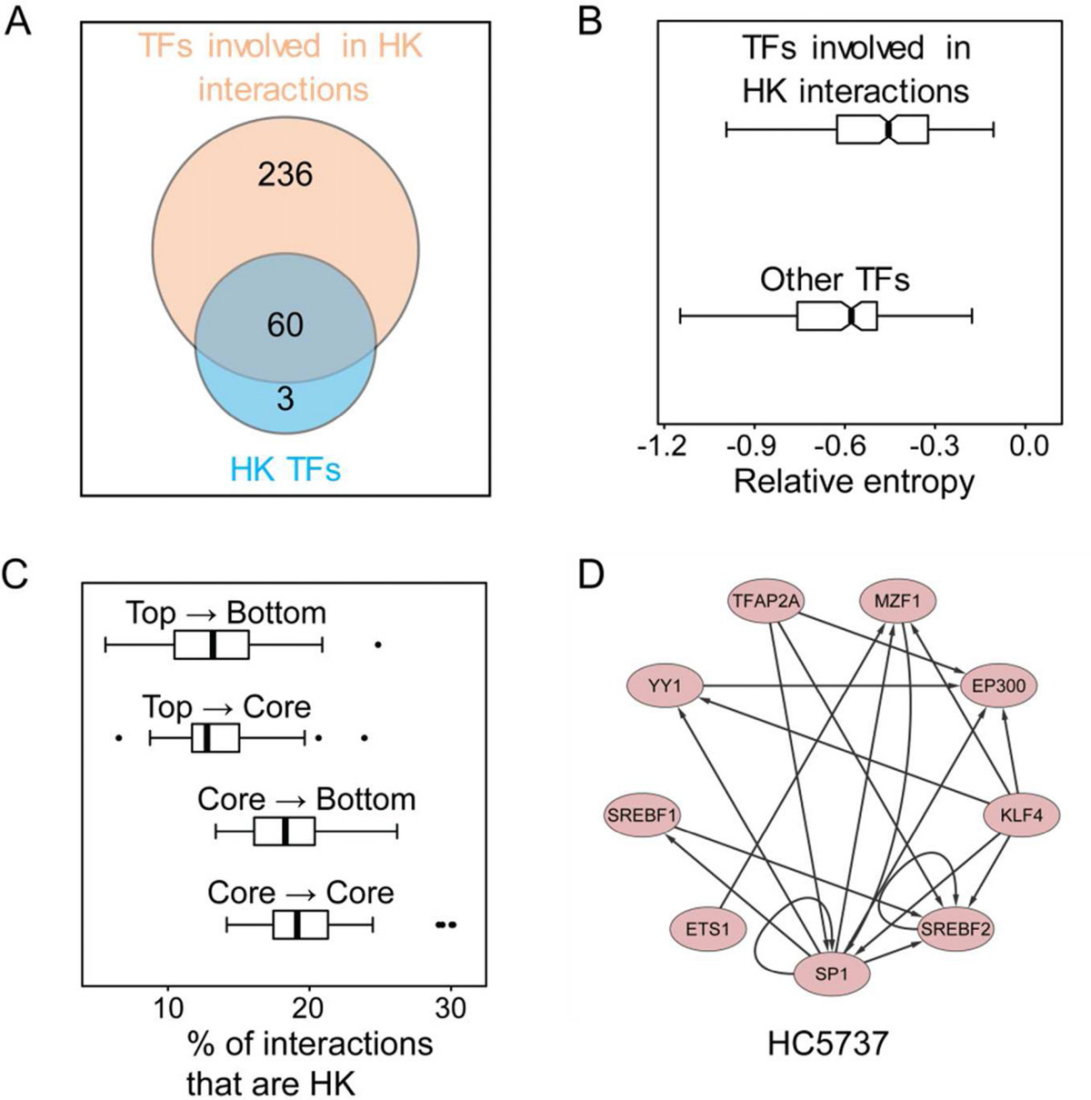
(**A**) The intersection of the subset of TFs that are involved in HK interactions and the subset of TFs that are encoded by HK genes. (**B**) The box plots of the relative entropy of the expression values of the genes encoding TFs involved in HK interactions (above) and other TFs (below). (**C**) The box plots of the proportions of HK interactions within the core layer and among the top, core and bottom layers in the 41 human cell-type TF regulatory networks. (**D**) TFs and HK interactions among them in a protein complex (id: HC5737) (24).

Neph *et al.* (18) remarked that ∼5% of all interactions (i.e. 2041 interactions) (Supplementary Table S5) are common across the 41 cell types. Our leave *k*-out validation shows that the number of common interactions in fewer cell types increases only slightly (Supplementary Figure S2). We therefore take these 2041 interactions as HK regulatory interactions. Enrichment analyses show that the proportions of HK links within the core layer and between the core and bottom layers are comparable and higher than those between the top and core layers and between the top and bottom layers (Figure 5C).

There are 296 TFs involved in HK interactions (Figure 5A). These TFs are not necessarily encoded by HK genes. But, as expected, they are enriched with TFs encoded by the HK genes listed in (30) (*P*-value = 1.27e−10; hypergeometric test). Additionally, the expressions of genes encoding them are much stabler than other TF genes across 79 human tissues (*P-*value = 4.32e−10) based on the entropy analysis of the gene expression data reported in (25) (Figure 5B). Similar results hold for the HK gene list obtained from combining the lists in (35–37) (Supplementary Figure S3).

### Regulatory interactions specific to hESCs

ESCs are derived from the inner cell mass of an early-stage embryo. Although OCT4, NANOG and other markers of hESCs have been identified, the whole picture of how TFs cooperate with each other in hESCs is largely unclear (38–40). There are 1509 regulatory interactions specific to hESCs, involving 411 TFs. The network induced by specific interactions over these TFs is referred to as the hESC-specific network (ESCSN). There are 82 hubs (the top 20% of the TFs with the largest total degree) (Table 1). Among the 82 hubs, only 35 are the hub TFs in the original hESC TF regulatory network. The remaining 47 hubs, including popular NANOG, seem to play unique roles in hESCs.

**Table 1:**
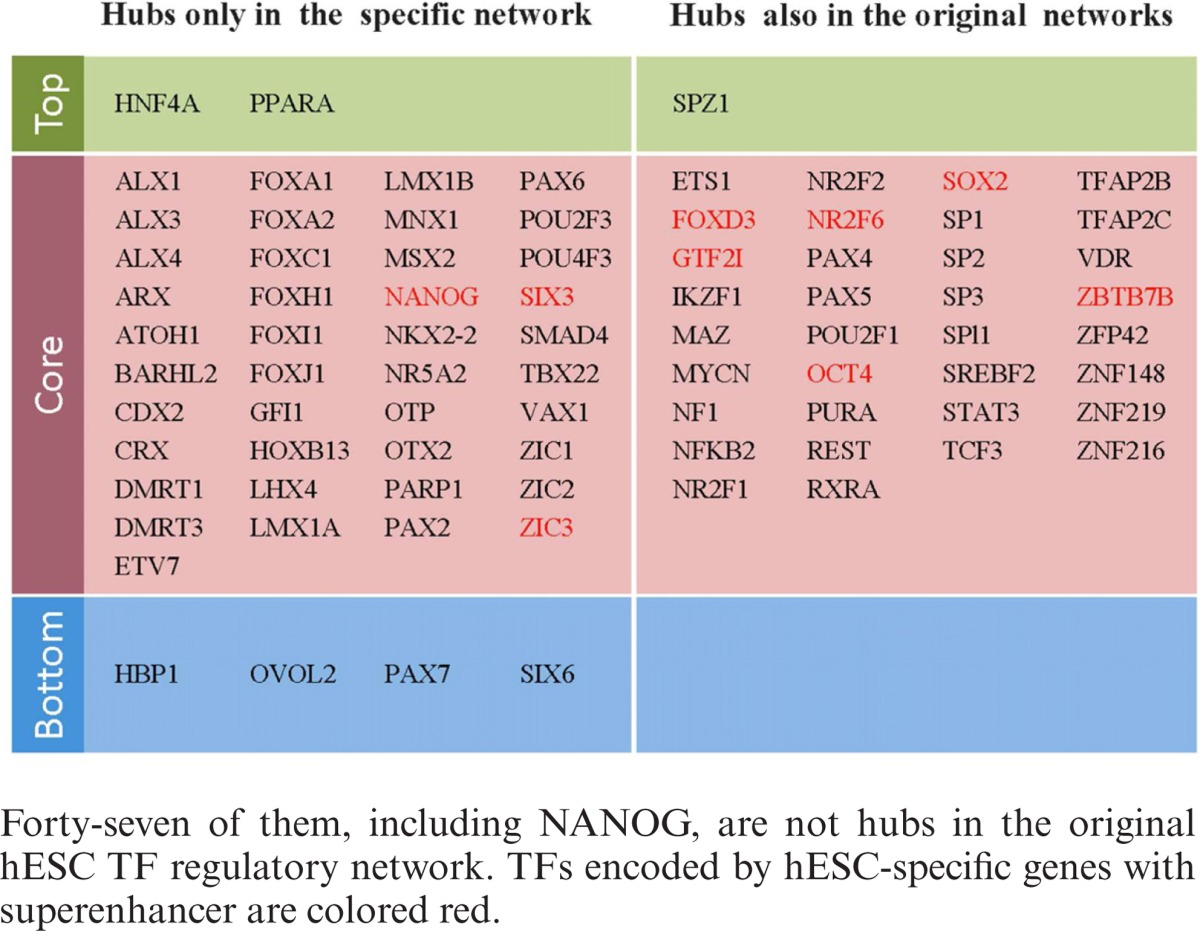
Eighty-two hub TFs in the ESCSN.

**Table 2:** The summary of the enrichment of hubs, essential and HK TFs in the top, core and bottom layers of the 41 cell-type TF regulatory networks.

	Hub TFs			Essential TFs			HK TFs		
	Top	Core	Bottom	Top	Core	Bottom	Top	Core	Bottom
Blood (7)	–	+	–			–	–	+	
Cancer (2)	–	+	–		+^c^	–^c^	–	+	
Endothelia (4)	–	+	–			–	–	+	
Epithelia (6)	–	+	–			–^b^	–	+	
ESC (1)		+	–		+	–			
Fetal (3)	–	+	–		+	–	–	+	
Stroma (14)	–^a^	+	–			–^a^	–	+	
Viscera (4)	–	+	–			–	–	+	

For clarity, the cell types are divided into eight classes, listed (together with the numbers of cell types) in the first column. The symbols + and – represent the enrichment and depletion of TFs of a type in a hierarchical layer in all the networks of a class.

^a^13 out of 14 are poor in hubs or essential TFs.

^b^Three out of six are poor in essential TFs.

^c^One out of two is enriched with or poor in essential TFs.

Superenhancers are large collections of transcriptional enhancers. Genes with superenhancer domain play important roles in the control of cell identity and diseases (41–43). In mouse and human ESCs, master TFs OCT4, SOX2, NANOG are each encoded by a gene with superenhancer and also have DNA-binding motifs that are often found in superenhancer domains (42). Most interestingly, nine hub TFs (colored red in Table 1) are each encoded by hESC-specific genes with superenhancer (*P*-value = 0.03; hypergeometric test) based on superenhancers reported in (41). They are FOXD3, GTF2I, NANOG, NR2F6, OCT4, SIX3, SOX2, ZBTB7B and ZIC3.

**Figure 6.**
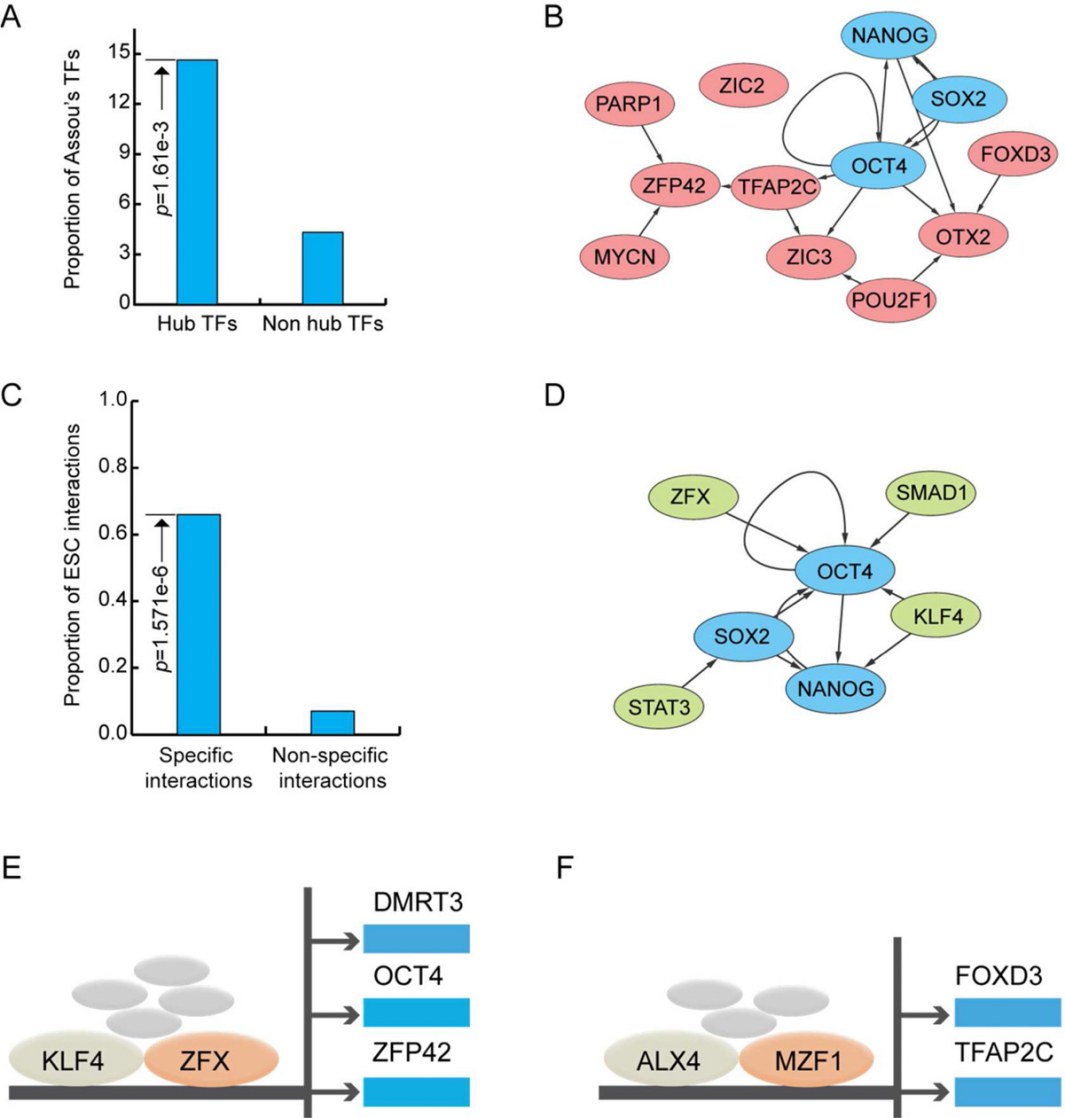
(**A**) Proportions of hub TFs that are in Assou *et al.*'s list (36) and the significance of their enrichment in the ESCSN. (**B**) The subnetwork induced by the hub TFs in the Assou *et al.*'s list in the ESCSN. (**C**) Proportions of known hESC interactions (38) and the significance of their enrichment in the ESCSN. (**D**) The hESC-specific regulatory interactions appearing in a reported core transcription network for hESCs (38). (**E**) and (**F**) Two specific regulatory complex-target modules in the hESCs.

Assou *et al.* (44) compiled a list of 1076 genes that are overexpressed in hESCs. In the ESCSN, the hubs are significantly enriched with the TFs encoded by the overexpressed genes in this list (*P*-value = 1.61e−3; hypergeometric test, Figure 6A). More interestingly, 12 of the hubs that are encoded by the genes in the list are well connected, except for ZIC2 (Figure 6B). NANOG, OTX2, PARP1, ZIC2 and ZIC3 are not hubs in the original hESC TF regulatory network.

ESCs self-renew indefinitely while maintaining pluripotency. Activin A is a member of the transforming growth factor β superfamily. It is found to play a central role in maintaining ‘stemness’ (45,46). Activin A initially binds to type II Activin A receptors and then recruits the Activin A receptor, type IB (ALK4). ALK4 further phosphorylates SMAD2/3. Upon activation by phosphorylation and association with SMAD4, SMAD2/3 translocates to the nucleus and upregulates the expression of other TF genes, such as *Oct4*, *Nanog*, *Modal*, *Wnt3* and *Fgf8*, and downregulates *Bmp*7 (46). In hESCs, SMAD3 tends to co-occupy DNA-binding sites with OCT4, SOX2 and NANOG in responses to transforming growth factor β signaling (47). The Nadal/Activin A signaling pathway is also enriched (false discovery rate = 9.86e−5) with the hubs in the ESCSN.

In addition, a core transcriptional regulatory network of hESCs (38) is enriched in hESC-specific interactions (*P*-value = 6.92e−6; hypergeometric test, Figure 6C), as shown in Figure 6D.

## DISCUSSION

We have studied the organizational architectures of the 41 human cell-type TF regulatory networks that were reported by Neph *et al.* (18). First, we have shown that the wiring around five to seven TFs in the networks can be used to classify all the 41 cell types well. Both Neph *et al.* (18) and our studies indicate that the human TF regulatory networks are different globally as well as locally.

Human regulatory networks exhibit hierarchical and modular structure (48). We have examined the three-layer hierarchical organizations of the human cell-type TF regulatory networks. The networks are each partitioned into the top, core and bottom layers, containing 23, 67 and 10% of TFs on average (Figure 3B, Supplementary Table S3), respectively. The large size and well-connectedness of the core layers are probably due to (i) master cell-type-specific TFs have a large number of target genes and (ii) their encoding genes have a superenhancer domain (41,42). For example, in the core layer of the hESC TF regulatory network, 326 TFs (81.3%) out of 401 are either the regulators or regulated by nine TFs each encoded by a gene with superenhancer domain, forming a large ‘bow-tie’ subnetwork (49).

The same hierarchical analysis (20) indicates that in the yeast TF regulatory networks, both the core and bottom layers have similar sizes (43 versus 40%), whereas the top layer contains only 13% of the TFs. Taken together, these two facts together imply a difference in the topological organizations between the human and yeast TF regulatory networks.

Enrichment analyses (Table 2) indicate that for each TF regulatory network of the 40 non-ESC cell types, (i) the top layer is lacking in both hub and HK TFs, (ii) the core layer is enriched with both hubs and HK TFs and (iii) the bottom layer is depleted with hub and essential TFs. However, essential TFs seem to be distributed evenly in the top and core layers, but, by and large, sparsely in the bottom layers.

Interestingly, the hESC TF regulatory network has a topological structure that is different from the rest. It has significantly small top and bottom layers and therefore a large core layer. Indeed, seven STATs and 15 key TFs (appearing in Figure 6B and D) are all found in the core layer. Moreover, 87.6% of links are within the core layer, whereas there are only 40 links (0.3%) between the top and bottom layers. These two facts together suggest that hESCs have a highly dense and well-connected TF regulatory network. And our analyses indicate that master TFs and superenhancer-associated TFs are in the kernel of the core layer. Its top layer is neither enriched with nor depleted of hub, essential and HK TFs, in contrast to the TF regulatory networks of the other cell types.

We have also studied the dynamic properties of the human cell-type TF regulatory networks. The HK interactions are related to basic life support such as biomolecular synthesis and transcription mechanisms. One of our findings is that most HK interactions are within the core layer or between the core and bottom layers. Using the identified HK interactions to investigate the protein complex database, we identified 23 protein complexes in which the proteins are highly connected with HK links (Supplementary Table S6). One of these complexes is given in Figure 5D. Most of the identified protein complexes are as predicted and hence it would be interesting to investigate their biological functions.

The ESCSN, the subnetwork induced by specific links in the hESC TF regulatory network, has also been investigated. The 82 hub TFs in the ESCSN (Table 1) seem to play important roles in hESCs due to the following facts: (i) their genes are overexpressed, (ii) they are enriched in the Activin A/Nodal signaling pathway and (iii) specific interactions are enriched in a core transcriptional regulatory network of the hESCs reported in (38). In general, specific regulatory interactions are difficult to detect because the network of each cell type is based on independent data, leading to a high false negative rate. Since the number of specific interactions in hESCs is much higher than that in other cell types, our results should not be greatly affected by the limitations of the data chosen.

Cell type specificity is believed to be the outcome of the interplay of the DNA sequence-binding specificity of TFs, cofactors and epigenetics (38,50). Through the integration of a database of protein complexes (24) and the ESCSN, we identified 55 hESC-specific regulatory complex-target modules (Methods, Supplementary Table S2). One of these modules is illustrated in Figure 6E: in a complex (id #: HC4463), both KLF4 and ZFX have three common downstream targets: FOXD3, OCT4 and ZFP42. As expected, KLF4, ZFX and their targets are important in the maintenance of pluripotency, self-renewal and development processes in ESCs (38,50–55). Another is given in Figure 6F, in which both ALX4 and MZF1 regulate FOXD3 and TFAP2C. Notably, FOXD3 has recently been demonstrated to be responsible in directing pluripotency and paraxial mesoderm fates in hESCs (56). All these facts together suggest that specific regulatory interactions may play important roles in hESCs.

## SUPPLEMENTARY DATA

Supplementary Data are available at NAR Online.

SUPPLEMENTARY DATA
