# Supplementary material for: Profiling the transcription factor regulatory networks of human cell types
Source: Nucleic Acids Res. 2014 Oct 9;42(20):12380–7. doi: 10.1093/nar/gku923 (PMC4227771; doi:10.1093/nar/gku923)
Supplement: SUPPLEMENTARY DATA [file supp_42_20_12380__index.html]

Profiling the transcription factor regulatory networks of human cell types — Profiling the transcription factor regulatory networks of human cell types — SUPPLEMENTARY DATA 

# Profiling the transcription factor regulatory networks of human cell types

## SUPPLEMENTARY DATA

**Files in this Data Supplement:**

- SUPPLEMENTARY DATA
- SUPPLEMENTARY DATA
