# Supplementary material for: Profiling the transcription factor regulatory networks of human cell types
Source: Nucleic Acids Res. 2014 Oct 9;42(20):12380–7. doi: 10.1093/nar/gku923 (PMC4227771; doi:10.1093/nar/gku923)
Supplement: SUPPLEMENTARY DATA [file supp_gku923_nar-01997-n-2014-File002.pdf]

Supplementary Document for

Profiling Human Cell-type Specific Transcription Factor Regulatory Networks

Shihua Zhang, Dechao Tian, Ngoc Hieu Tran, Kwok Pui Choi, Louxin Zhang

Supplementary figures

Figure S1
Figure S2
Figure S3

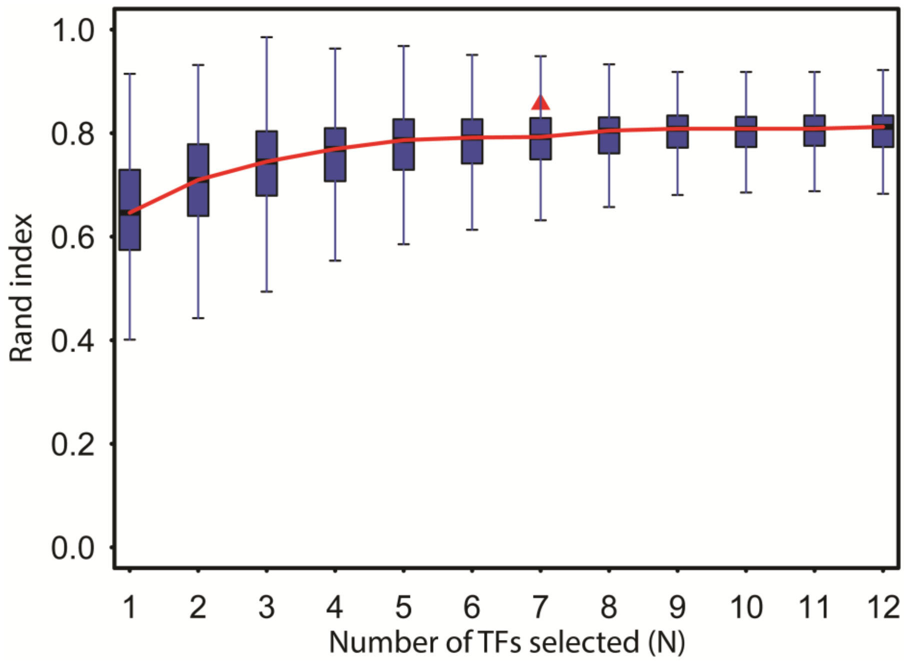

Figure S1 The boxplots of the Rand Index values for the classifications of the 41 cell types using N randomly selected TFs. The number of repetitions taken was 1000 for each N. See the Methods section for details.

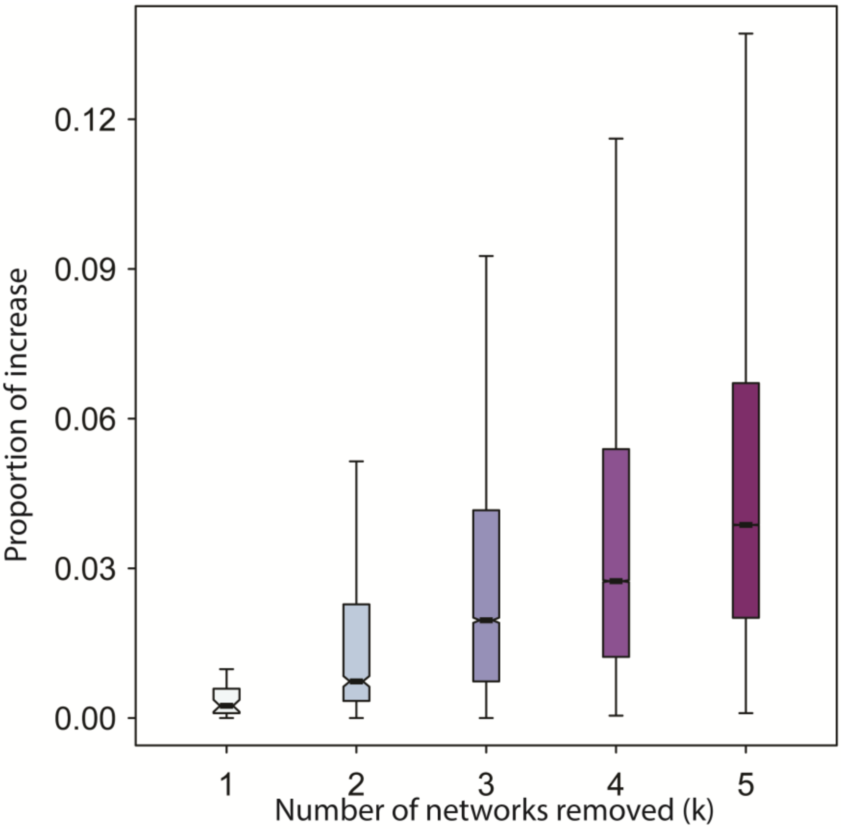

Figure S2 **The b**oxplots for the proportional increase for the leave-*k*-out validation. For $k = 1, 2, \ldots, 5$, we compute the proportional increase, $y/2041 - 1$, where y is the number of regulatory interactions that appear in the remaining (41-*k*) networks after the *k* selected networks are removed.

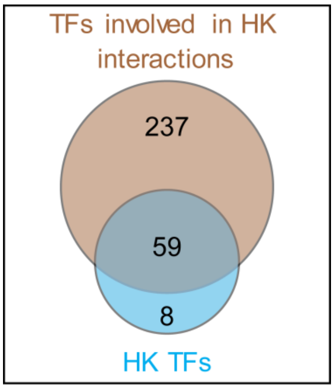

Figure S3 The set of 296 TFs involved in the HK interactions are enriched with 67 TFs that are encoded by HK genes given in the references 29-31.
